# Supplementary material for: Using induced pluripotent stem cells to investigate human neuronal phenotypes in 1q21.1 deletion and duplication syndrome
Source: Mol Psychiatry. 2021 Jun 10;27(2):819–30. doi: 10.1038/s41380-021-01182-2 (PMC9054650; doi:10.1038/s41380-021-01182-2)
Supplement: Supplementary file 1 — Supplementary information [file 41380_2021_1182_MOESM1_ESM.docx]

**Supplementary Methods**

## Calcium Imaging

Day 40 neuronal differentiations were passaged onto poly-d-lysine and laminin coated coverslips at a density of 50,000 cells/cm^2^. For pharmacological interventions, drugs (50nM verapamil and 2.5µM roscovitine) were applied 1 day after passaging and concentrations were maintained during all media changes (every 3 days). After 6 days media was replaced with BPM (BrainPhys™ Neuronal Medium supplemented with B27+ supplement, penicillin, streptomycin, glutamine, 10ng/mL BDNF and 35 µg/mL ascorbic acid). After 50 days of differentiation neurons were loaded with 1µM Cal-520® (AAT Bioquest) for 1 hour in BPM containing 0.02% Pluronic F127. The media was then replaced with fresh BPM and cells were incubated for a further 1 hour at 37°C. Coverslips were transferred into artificial cerebrospinal fluid (aSCF) containing: 125mM NaCl, 26mM NaHCO_3_, 1.25mM KH_2_PO_4_, 2.5mM KCl, 1mM MgCl_2_, 2mM CaCl_2_ and 25mM Glucose with or without either DL-AP5 or NQBX (at a final concentration of 10µM). Images were taken using an epifluorescence microscope at intervals of 200ms for 5 minutes. A minimum of 3 technical replicates (separate image stacks from a single culture) were averaged to generate each data point. Each technical replicate had 3 wells. A minimum of 6 random field per well was chosen, with each field containing 80-120 active cells. Images were analysed using NeuroCa and Matlab. After automated analysis events with a rise time or fall time of more than 2 seconds were discarded and for the purpose of analysing average number of events all cells with no events were also discarded.

## Multiple Electrode Arrays

All experiments were performed using CytoView MEA 24-well plates (M384-tMEA-24W). MEAs were first pre-treated with 0.01% polyethylenimine (Sigma) and incubated for 1 hour at 37°C. Day 50 neurons were plated as high density drop cultures (5,000 cells/µL) containing 10µg/ml laminin. After 1-hour, conditioned medium was added into each MEA and after 24 hours 0.5ml of fresh BPM was added to each array. MEA cultures were maintained in 1:1 fresh to astrocyte condition BPM replaced every 3-4 days. Electrophysiological activity was recorded every 10 days using hardware (Maestro Pro complete with Maestro 768-channel amplifier) and software (AxIS 1.5.2) from Axion Biosystems (Axion Biosystems Inc., Atlanta, GA). Channels were sampled simultaneously with a gain of 1000× and a sampling rate of 12.5 kHz/channel. During the recording, the temperature was maintained constant at 37°C. A Butterworth band-pass filter (with a high-pass cut-off of 200 Hz and low-pass cut-off of 3000Hz) was applied along with a variable threshold spike detector set at 5.5× standard deviation on each channel. Offline analysis was achieved with custom scripts written in MATLAB (available on request). Briefly, spikes were detected from filtered data using an automatic threshold-based method set at -5.5 x σ, where σ is an estimate of the noise of each electrode based upon the median absolute deviation 1. Spike timestamps were analysed to provide statistics on the general excitability of cultures. Neuronal bursting was detected based on three parameters: inter-burst period longer than 200ms, more than 3 spikes in each burst and a maximum inter-spike (intra-burst) interval of 300 milliseconds. Network activity was illustrated by creating array–wide spike detection rate (ASDR) plots with a bin width of 200 ms. Synchronised bursts (SBs) across all electrodes in the network were identified using Axion built-in neural metric analysis tool employing the envelope algorithm. The algorithm defines a SB by identifying times when the histogram exceeds a threshold of 1 standard deviation above or below the mean with a minimum of 200ms between SBs and at least 10% of electrodes included. All active electrodes were included in the analysis. A minimum of three MEAs/cell line/differentiation have been considered for the analysis.

**Gene expression analysis**

RNA was isolated using a GeneElute™ mammalian total RNA miniprep kit (Sigma). A minimum of 500ng of RNA was used to create cDNA using a high-capacity cDNA reverse transcription kit (Applied Biosystems). For quantitative real-time PCR 100ng of cDNA was used per reaction and primers sequences used are listed in Supplementary Table 2. Reactions were run on a StepOnePlus™ real-time PCR system (Applied Biosystems) with qPCRBIO SyGreen Blue Mix Hi-ROX (PCR Biosystems) PCR products were detected by incorporation of SYBR-green and authenticated by the melt-curves. Result were normalised to GAPDH are presented as either relative expression calculated by the 2^−ΔΔCT^ method or by relative mRNA abundance. All samples were run in triplicate (technical replicates) and averaged before further data analysis.

**Western Blotting**

Cell lysis was performed in RIPA buffer containing protease and phosphatase inhibitors (MS-SAFE, Sigma). Protein concentration was quantified using the *DC*™ protein assay (Biorad). The samples were run on Bis-Tris 4-12% gradient gels (Thermo Fisher) and proteins were transferred to a nitrocellulose membrane. Membranes were blocked using 5% milk in tris-buffered saline (TBS) with Tween 20 (TBST). Primary antibodies (Supplementary Table 3) were diluted in blocking solution and then applied to the membranes over night at 4°C. Secondary antibodies (IRDye, Li-Cor) were applied in blocking solution for 1-1.5 hours at room temperature. Membranes were visualized using the Odessay CLx (LiCor). Quantification of bands was done using Image Studio (LiCor) and all results were normalized to GAPDH. Samples were run in triplicate (technical replicates) and were average before further data analysis.

**Immunofluorescence and cell counting**

Cells were fixed using 4% paraformaldehyde then blocked and permeabilised using 5% donkey or goat serum in PBS with 0.01-0.3% triton-X-100 (PBST). Primary antibodies (Supplementary Table 3) were applied in blocking solution and incubated overnight at 4°C. Secondary antibodies were diluted in PBST and applied for 1 hour at room temperature. Cells were counterstaining with DAPI and mounted using ProLong™ Glass Antifade Mountant (Thermo Fisher). A minimum of 3 random fields (each field containing a minimum of 20 cells) were chosen for quantification per biological replicate. Quantification was carried out using CellProfiler^1^ to quantify positivity compared to DAPI. For nuclear staining, positive cells were counted only if staining overlapped with DAPI and for cytoplasmic staining positive cells were counted if staining was found in contact with DAPI. To quantify pre-synaptic puncta Intellicount ^2^ was used to compare synaptic puncta to MAP2+ area using the machine learning function and an object size maxima of 9µm^2^. All image analysis was performed on images taken using a Leica DMI600B inverted time-lapse microscope with a 20x objective.

**Histological analysis of mice brains**

Perfused and fixed brains from one month old mice modelling 1q21.1 microdeletion^3^ and wild type littermates were purchased from Taconic USA (11025, n=6). Brains were incubated in 30% (wt/wt) sucrose before mounting in O.C.T (Fisher Scientific). Tissue was sliced on a cryostat at 10µm. Antigen retrieval was performed using citrate buffer. Sections were blocked and permeabilised using a solution of 5% donkey serum in PBST (with 0.1-0.5% triton-x-100). Primary antibodies (Supplementary Table 3) were applied in blocking solution overnight at 4°C. Secondary antibodies were diluted in PBST and applied for 1 hour at room temperature. Tissue was counterstained with DAPI and mounted using ProLong™ Glass Antifade Mountant (Thermo Fisher). A minimum of three sections per brain were chosen for quantification (technical replicates) and quantification was carried out using CellProfiler comparing Tbr1+ or Ctip2+ cells to DAPI in a predetermined area.

**Supplementary Fig. 1** **Characterization of iPSCs generated from 1q21.1 deletion patient 1. A** Representative image of iPSCs stained for 3 markers of pluripotency (SOX2, OCT4 and NANOG). **B** Expression of OCT4 in iPSCs generated from 1q21.1 deletion patient 1 as compared to a positive control (hESCs) and a negative control (control iPSC derived neurons). **C** Expression of SOX2 in iPSCs generated from 1q21.1 deletion patient 1 as compared to a positive control (hESCs) and a negative control (control iPSC derived neurons). **D** Expression of C-MYC in iPSCs generated from 1q21.1 deletion patient 1 as compared to a positive control (hESCs) and a negative control (control iPSC derived neurons). **E** Expression of KLF4 in iPSCs generated from 1q21.1 deletion patient 1 as compared to a positive control (hESCs) and a negative control (control iPSC derived neurons). **F** Representative images and gene expression of SOX17 in iPSCs pushed to an endoderm fate. **G** Representative images and gene expression of BRACHYURY in iPSCs pushed to a mesoderm fate. **H** Representative images and gene expression of PAX6 in iPSCs pushed to an ectoderm fate. All data is presented as mean ± SEM, (n≥3) and where appropriate data was analysed by Students T-Test: ****P<0.0001 vs negative control. Scale

bar = 100µm

**Supplementary Fig. 2** **Characterization of iPSCs generated from 1q21.1 deletion patient 2. A** Representative images of IPSCs stained for 3 markers of pluripotency (SOX2, OCT4 and NANOG). **B** Expression of OCT4 in iPSCs generated from 1q21.1 deletion patient 2 as compared to a positive control (hESCs) and a negative control (control iPSC derived neurons). **C** Expression of SOX2 in iPSCs generated from 1q21.1 deletion patient 2 as compared to a positive control (hESCs) and a negative control (control iPSC derived neurons). **D** Expression of C-MYC in iPSCs generated from 1q21.1 deletion patient 2 as compared to a positive control (hESCs) and a negative control (control iPSC derived neurons). **E** Expression of KLF4 in iPSCs generated from 1q21.1 deletion patient 2 as compared to a positive control (hESCs) and a negative control (control iPSC derived neurons). **F** Representative images and gene expression of SOX17 in iPSCs pushed to an endoderm fate. **G** Representative images and gene expression of BRACHYURY in iPSCs pushed to a mesoderm fate. **H** Representative images and gene expression of PAX6 in iPSCs pushed to an ectoderm fate. All data is presented as mean ± SEM, (n≥3) and where appropriate data was analysed by Students T-Test: ****P<0.0001 vs negative control. Scale

bar = 100µm

**Supplementary Fig. 3** **Characterization of iPSCs generated from 1q21.1 deletion patient 3. A** Representative images of IPSCs stained for 3 markers of pluripotency (SOX2, OCT4 and NANOG). **B** Expression of OCT4 in iPSCs generated from 1q21.1 deletion patient 3 as compared to a positive control (hESCs) and a negative control (control iPSC derived neurons). **C** Expression of SOX2 in iPSCs generated from 1q21.1 deletion patient 3 as compared to a positive control (hESCs) and a negative control (control iPSC derived neurons). **D** Expression of C-MYC in iPSCs generated from 1q21.1 deletion patient 3 as compared to a positive control (hESCs) and a negative control (control iPSC derived neurons). **E** Expression of KLF4 in iPSCs generated from 1q21.1 deletion patient 3 as compared to a positive control (hESCs) and a negative control (control iPSC derived neurons). **F** Representative images and gene expression of SOX17 in iPSCs pushed to an endoderm fate. **G** Representative images and gene expression of BRACHYURY in iPSCs pushed to a mesoderm fate. **H** Representative images and gene expression of PAX6 in iPSCs pushed to an ectoderm fate. All data is presented as mean ± SEM, (n≥3) and where appropriate data was analysed by Students T-Test: ****P<0.0001 vs negative control. Scale

bar = 100µm

**Supplementary Fig. 4** **Characterization of iPSCs generated from 1q21.1 duplication patient 1. A** Representative images of IPSCs stained for 3 markers of pluripotency (SOX2, OCT4 and NANOG). **B** Expression of OCT4 in iPSCs generated from 1q21.1 duplication patient 1 as compared to a positive control (hESCs) and a negative control (control iPSC derived neurons). **C** Expression of SOX2 in iPSCs generated from 1q21.1 duplication patient 1 as compared to a positive control (hESCs) and a negative control (control iPSC derived neurons). **D** Expression of C-MYC in iPSCs generated from 1q21.1 duplication patient 1 as compared to a positive control (hESCs) and a negative control (control iPSC derived neurons). **E** Expression of KLF4 in iPSCs generated from 1q21.1 duplication patient 1 as compared to a positive control (hESCs) and a negative control (control iPSC derived neurons). **F** Representative images and gene expression of SOX17 in iPSCs pushed to an endoderm fate. **G** Representative images and gene expression of BRACHYURY in iPSCs pushed to a mesoderm fate. **H** Representative images and gene expression of PAX6 in iPSCs pushed to an ectoderm fate. All data is presented as mean ± SEM, (n≥3) and where appropriate data was analysed by Students T-Test: ****P<0.0001 vs negative control. Scale bar = 100µm

**Supplementary Fig. 5** **Characterization of iPSCs generated from 1q21.1 duplication patient 2. A** Representative image of IPSCs stained for 3 markers of pluripotency (SOX2, OCT4 and NANOG). **B** Expression of OCT4 in iPSCs generated from 1q21.1 duplication patient 2 as compared to a positive control (hESCs) and a negative control (control iPSC derived neurons). **C** Expression of SOX2 in iPSCs generated from 1q21.1 duplication patient 2 as compared to a positive control (hESCs) and a negative control (control iPSC derived neurons). **D** Expression of C-MYC in iPSCs generated from 1q21.1 duplication patient 2 as compared to a positive control (hESCs) and a negative control (control iPSC derived neurons). **E** Expression of KLF4 in iPSCs generated from 1q21.1 duplication patient 2 as compared to a positive control (hESCs) and a negative control (control iPSC derived neurons). **F** Representative images and gene expression of SOX17 in iPSCs pushed to an endoderm fate. **G** Representative images and gene expression of BRACHYURY in iPSCs pushed to a mesoderm fate. **H** Representative images and gene expression of PAX6 in iPSCs pushed to an ectoderm fate. All data is presented as mean ± SEM, (n≥3) and where appropriate data was analysed by Students T-Test: ****P<0.0001 vs negative control. Scale bar = 100µm

**Supplementary Fig. 6** **Gene expression of 1q21.1 gene**. **A** Schematic plot of the 1q21.1 locus. This CNV spans ~3Mb and comprises of two regions (TAR and Distal), genes known to be involved in the 1q21.1 distal/critical region (1.35Mb) are illustrated. **B** Bar graph showing mRNA expression changes of key genes within the 1q21.1 distal region in iPSC derived cortical neurons following 50 days of differentiation. **C** mRNA expression of NOTCH2NL after 20 days of neuronal differentiation. Data was analysed using Students T-Tests. All data presented as means ± SEM *P<0.05; **P<0.01 vs. control.

**Supplementary Fig. 7 Characterization of iPSC derived neurons with and without 1q21.1 CNV**. **A** The expression of PAX6, PLZF and ZO1 mRNA at day 20 of neuronal differentiation in control lines. The time point samples were taken had a significant effect on expression of the three markers (Control n=3). **B** Fold change of NPC markers in 1q21.1 deletion and duplication cell lines after 20 days of differentiation. Data was analysed using multiple T-Tests. Stars represent Holm-Sidak corrected p-values. **C, D** Expression of Ki67 mRNA after 20 and 30 days of differentiation (d20, d30; n=3). **E** The expression of DCX, NCAM and MAP2 mRNA at day 20 and 50 of neuronal differentiation. The time point samples were taken had a significant effect on expression of the three markers (Control n=3; F1,30=98.4; P<0.0001). Data sets were analyzed by two-way ANOVA with post hoc comparisons comparing to day 20 samples. Stars above points represent Sidak-corrected post hoc tests. **F** Fold change of neuronal markers in 1q21.1 deletion and duplication cell lines after 50 days of differentiation. Data was analyzed using multiple T-Tests. Stars represent Holm-Sidak corrected p-values. **G** Expression of cortical layer markers in control day 50 cultures (n=3). **H** Expression of cortical layer markers in 1q21.1 deletion and duplication day 50 cultures (n=3). Fold change is normalized to control day 50 differentiations. Data was analyzed using multiple t-Tests. Stars represent Holm-Sidak corrected p-values. All data presented as means ± SEM *P<0.05; ***P<0.001 ****P<0.0001 vs. control. **I** The expression of GFAP and S100β was significantly minimal in comparison to MAP2 in D40 neuronal cultures and the level of GFAP and S100β expression was comparable across the experimental group. **J** Similarly, the expression of GFAP and S100β in comparison to MAP2 was significantly reduced in D50 neuronal cultures (n=3). Data was analysed using Students T-Tests. **K** Bar graph showing level of TUJ1 expression across group at day 50. All data presented as means ± SEM, n=3, *P<0.05, One way ANOVA, Dunnet multiple comparison between group was not significant.  **L-N** Representative confocal fluorescence images of neurons across groups co-immunostained for MAP2 (green) and TUJ1 (red) after 50days of differentiation. Nuclei were counterstained by DAPI (blue). Scale bar: 50μm.

**Supplementary Fig. 8 Morphological characterization of 1q21.1 immature neurons**. **A** Quantification of average soma size of neurons after 30 days of differentiation (n≥3). **B** Quantification of MAP2+ process length of neurons after 30 days of differentiation (n≥3). **C** Quantification of the number of primary MAP2+ branches of neurons after 30 days of differentiation (n≥3). Data was analysed using Students T-Tests. All data presented as means ± SEM *P<0.05; ***P<0.001 ****P<0.0001 vs. control.

**Supplementary Fig. 9 Expression of PSD-95 is reciprocally affected by 1q21.1 mutation**. **A** The expression of synaptophysin protein at day 50 of neuronal differentiation normalized to GAPDH (n≥3). **B** Histogram of PSD-95 expression normalised to GAPDH (n≥3). Data was analyzed using Students T-Tests and all data is presented as means ± SEM; *P<0.05, ***P<0.001, ****P<0.0001 vs Control. **C** Representative western blots of GAPDH, PSD-95 and MAP2 from a Control, Deletion and Duplication sample.

**Supplementary Fig. 10 Inhibition of AMPA or NMDA signalling results in decreased neuronal function.** **A** Quantification of soma which show at least 1 characteristically neuronal calcium event (n≥3). Both genotype (F2,19=97.44; P<0.0001; n≥3/group) and drug (F2,19=100.5; P<0.05; n≥3/group) had significant effects on the percentage of active cells. There wasalso a significant interaction between the effect of genotype and drug (F4,19=10.16; P=0.0001; n≥3/group) on the percentage of active cells. Data sets were analysed by two-way ANOVA with post hoc comparisons using Dunnett’s multiple comparisons test comparing to control samples. Stars represent Dunnett-corrected post hoc tests. All data presented as means ± SEM *P<0.05; ****P<0.0001 vs. untreated. **B** Example of an array-wide spike detection rate (ASDR) plot from 1q21.1 deletion cultures after 100 days of differentiation. **C** Example of an array-wide spike detection rate (ASDR) plot from 1q21.1 deletion cultures after 100 days of differentiation. The culture was incubated with AP5 immediatly before recording. **D** Example of an array-wide spike detection rate (ASDR) plot from 1q21.1 deletion cultures after 100 days of differentiation. The culture was incubated with CNQX immediatly before recording. **E** Representative raster plot of neuronal activity exhibited by control and duplication-derived neurons at early (D60) neurodevelopmental stage. **F** The average number of spikes recorded per electrode at D60 of differentiation in control and duplication-derived neurons, there was no significant difference between the spike counts in both models.

**Supplementary Fig. 11 Gene expression of ion channels in control day 50 neurons.** Expression of common neuronal ion channels in day 50 control (average of both control, n≥3) neuronal cultures normalised to the expression of GAPDH.

**Reference:**

1. Carpenter AE, Jones TR, Lamprecht MR, Clarke C, Kang IH, Friman O *et al.* CellProfiler: image analysis software for identifying and quantifying cell phenotypes. *Genome biology* 2006; **7**(10)**:** R100.

2. Fantuzzo JA, Mirabella VR, Hamod AH, Hart RP, Zahn JD, Pang ZP. Intellicount: High-Throughput Quantification of Fluorescent Synaptic Protein Puncta by Machine Learning. *eNeuro* 2017; **4**(6).

3. Nielsen J, Fejgin K, Sotty F, Nielsen V, Mork A, Christoffersen CT *et al.* A mouse model of the schizophrenia-associated 1q21.1 microdeletion syndrome exhibits altered mesolimbic dopamine transmission. *Transl Psychiatry* 2017; **7**(11)**:** 1261.
