# Supplementary material for: Using induced pluripotent stem cells to investigate human neuronal phenotypes in 1q21.1 deletion and duplication syndrome
Source: Mol Psychiatry. 2021 Jun 10;27(2):819–30. doi: 10.1038/s41380-021-01182-2 (PMC9054650; doi:10.1038/s41380-021-01182-2)
Supplement: Supplementary file 6 — Supplementary Figure 5 [file 41380_2021_1182_MOESM6_ESM.pdf]

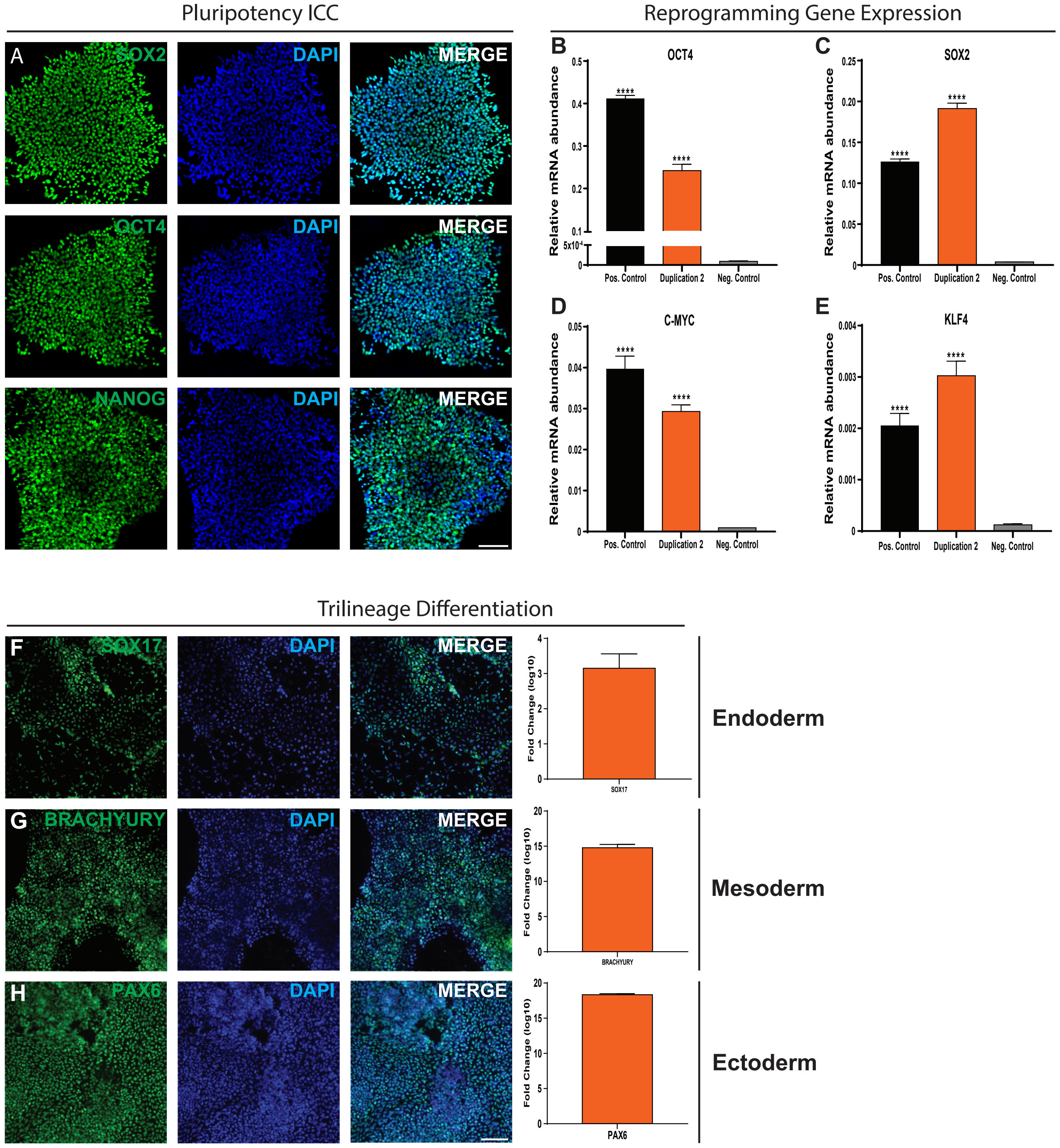


**Supp. Fig. 5: Characterization of iPSCs generated from 1q21.1 duplication patient 2. A** Representative images of IPSCs stained for 3 markers of pluripotency (SOX2, OCT4 and NANOG). **B** Expression of OCT4 in iPSCs generated from 1q21.1 duplication patient 2 as compared to a positive control (hESCs) and a negative control (control iPSC derived neurons). **C** Expression of SOX2 in iPSCs generated from 1q21.1 duplication patient 2 as compared to a positive control (hESCs) and a negative control (control iPSC derived neurons). **D** Expression of C-MYC in iPSCs generated from 1q21.1 duplication patient 2 as compared to a positive control (hESCs) and a negative control (control iPSC derived neurons). **E** Expression of KLF4 in iPSCs generated from 1q21.1 duplication patient 2 as compared to a positive control (hESCs) and a negative control (control iPSC derived neurons). **F** Representative images and gene expression of SOX17 in iPSCs pushed to an endoderm fate. **G** Representative images and gene expression of BRACHYURY in iPSCs pushed to a mesoderm fate. **H** Representative images and gene expression of PAX6 in iPSCs pushed to an ectoderm fate. All data is presented as mean ± SEM, (n≥3) and where appropriate data was analysed by students T-Test: ****P<0.0001 vs negative control. Scale bar = 100μm.
