# Supplementary material for: Using induced pluripotent stem cells to investigate human neuronal phenotypes in 1q21.1 deletion and duplication syndrome
Source: Mol Psychiatry. 2021 Jun 10;27(2):819–30. doi: 10.1038/s41380-021-01182-2 (PMC9054650; doi:10.1038/s41380-021-01182-2)
Supplement: Supplementary file 7 — Supplementary Figure 6 [file 41380_2021_1182_MOESM7_ESM.pdf]

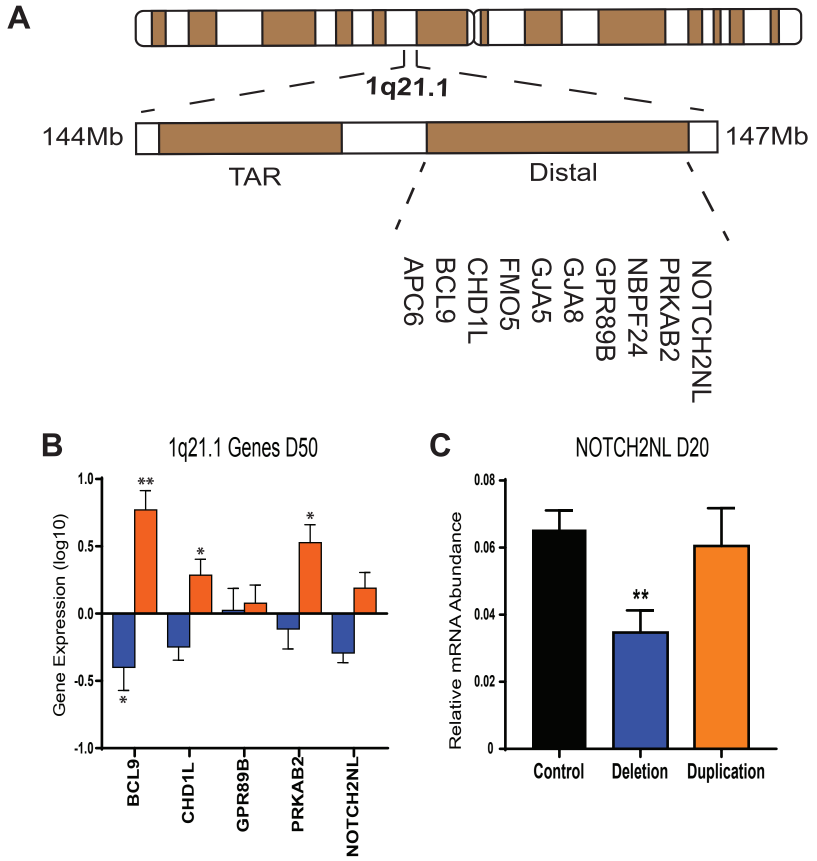

Suppl. Fig. 6: Gene expression of 1q21.1 genes. A Schematic plot of the 1q21.1 locus. This CNV spans ~3Mb and comprises of two regions (TAR and Distal), genes known to be involved in the 1q21.1 distal/critical region (1.35Mb) are illustrated. **B** Bar graph showing mRNA expression changes of key genes within the 1q21.1 distal region in iPSC derived cortical neurons following 50 days of differentiation. **C** mRNA expression of NOTCH2NL after 20 days of neuronal differentiation. Data was analysed using Students T-Tests. All data presented as means ± SEM *P<0.05; **P<0.01 vs. control.
