# Supplementary material for: Using induced pluripotent stem cells to investigate human neuronal phenotypes in 1q21.1 deletion and duplication syndrome
Source: Mol Psychiatry. 2021 Jun 10;27(2):819–30. doi: 10.1038/s41380-021-01182-2 (PMC9054650; doi:10.1038/s41380-021-01182-2)
Supplement: Supplementary file 8 — Supplementary Figure 7 [file 41380_2021_1182_MOESM8_ESM.pdf]

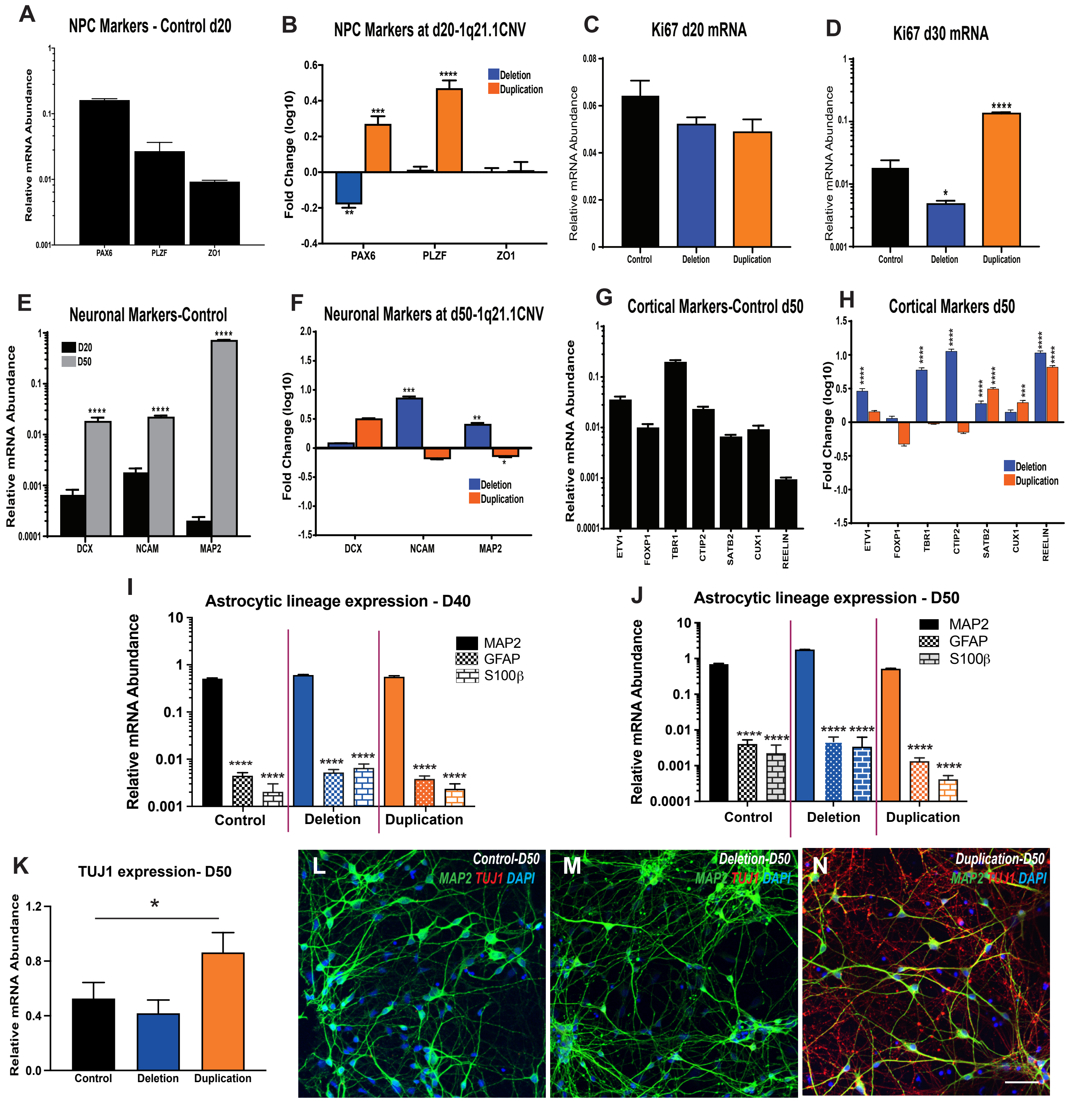

Supp. Fig. 7: Characterization of iPSC derived neurons with and without 1q21.1 CNV . A The expression of NESTIN (NES), PLZF and ZO1 mRNA at day 20 and 50 of neuronal differentiation. The time point samples were taken had a significant effect on expression of the three markers (Control n=3, $F_{1,30}$=23.2; P<0.0001;). **B** Fold change of NPC markers in 1q21.1 deletion and duplication cell lines after 20 days of differentiation. Data was analyzed using multiple T-Tests. Stars represent Holm-Sidak corrected p-values. **C,D** Expression of KI67 mRNA after 20 and 30 days of neuronal differentiation (n≥3).**E** The expression of DCX, NCAM and MAP2 mRNA at day 20 and 50 of neuronal differentiation. The time point samples were taken had a significant effect on expression of the three markers (Control n=3; $F_{1,30}$=98.4; P<0.0001). Data sets were analyzed by two-way ANOVA with post hoc comparisons comparing to day 20 samples. Stars above points represent Sidak-corrected post hoc tests. **F** Fold change of neuronal markers in 1q21.1 deletion and duplication cell lines after 50 days of differentiation. Data was analyzed using multiple T-Tests. Stars represent Holm-Sidak corrected p-values. **G** Expression of cortical layer markers in control day 50 cultures (n=3). **H** Expression of cortical layer markers in 1q21.1 deletion and duplication day 50 cultures (n=3). Fold change is normalized to control day 50 differentiations. Data was analyzed using multiple T-Tests. Stars represent Holm-Sidak corrected p-values. All data presented as means ± SEM, *P<0.05, ***P<0.001, ****P<0.0001. **I** The expression of GFAP and S100β was significantly minimal in comparision to MAP2 in D40 neuronal cultures. The level of GFAP and S100β was comparable across the experimental group. **J** Similarly, the expression of GFAPand S100β in compartision to MAP2 was signficantly reduced in D50 neuronal cultures (n=3). Data was analysed using Students T-Tests. **K** Bar graph showing level of TUJ1 expression across group at day 50. All data presented as means ± SEM, n=3, *P<0.05, One way ANOVA, Dunnet multiple comparison between group was not significant. **L-N** Representative confocal fluorescence images of neurons across groups co-immunostained for MAP2 (green) and TUJ1 (red) after 50days of differentiation. Nuclei were counterstained by DAPI (blue). Scale bar: 50μm.
