# Supplementary material for: Using induced pluripotent stem cells to investigate human neuronal phenotypes in 1q21.1 deletion and duplication syndrome
Source: Mol Psychiatry. 2021 Jun 10;27(2):819–30. doi: 10.1038/s41380-021-01182-2 (PMC9054650; doi:10.1038/s41380-021-01182-2)
Supplement: Supplementary file 9 — Supplementary Figure 8 [file 41380_2021_1182_MOESM9_ESM.pdf]

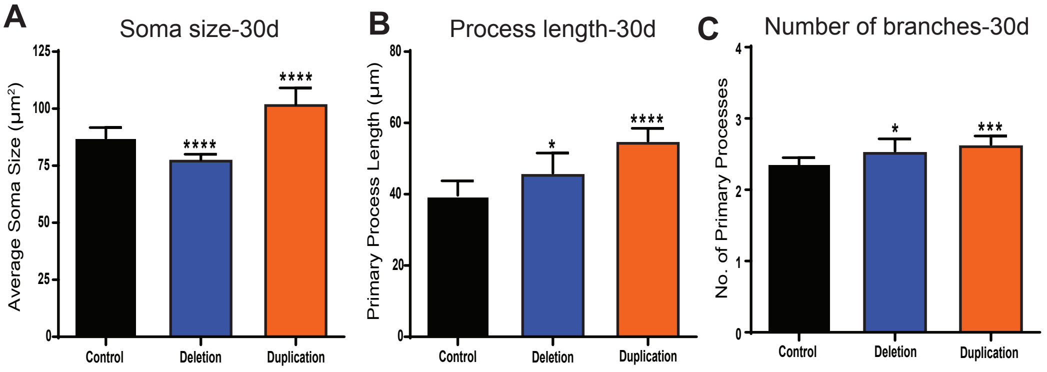


**Supp. Fig. 8: Mophological characterization of 1q21.1 immature neurons. A** Quantification of average soma size of neurons after 30 days of differentiation (n≥3). **B** Quantification of MAP2+ process length of neurons after 30 days of differentiation (n≥3). **C** Quantification of the number of primary MAP2+ branches of neurons after 30 days of differentiation (n≥3). Data was analysed using Students T-Tests. All data presented as means ± SEM *P<0.05; ***P<0.001 ****P<0.0001 vs. control.
