# Supplementary material for: Using induced pluripotent stem cells to investigate human neuronal phenotypes in 1q21.1 deletion and duplication syndrome
Source: Mol Psychiatry. 2021 Jun 10;27(2):819–30. doi: 10.1038/s41380-021-01182-2 (PMC9054650; doi:10.1038/s41380-021-01182-2)
Supplement: Supplementary file 10 — Supplementary Figure 9 [file 41380_2021_1182_MOESM10_ESM.pdf]

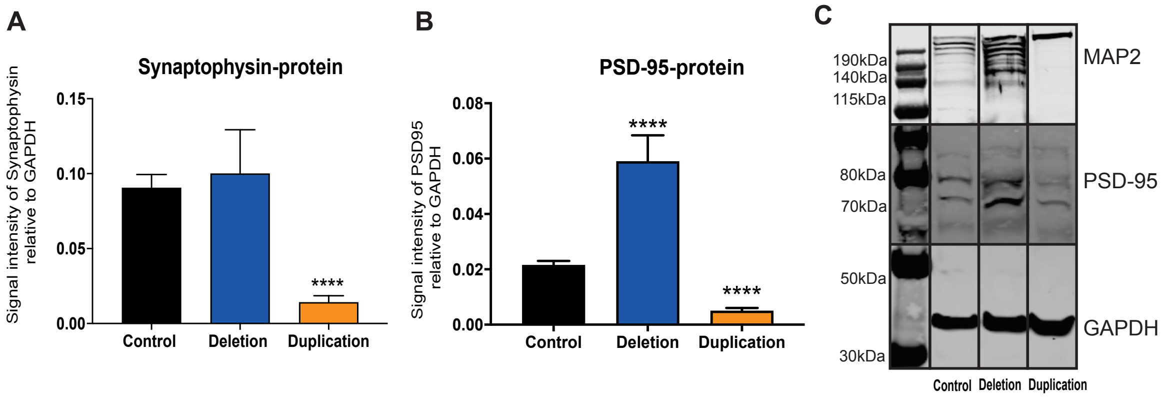


**Supp. Fig. 9: Expression of Synaptophysin and PSD-95 in iPSC derived neurons with1q21.1 CNV. A** The expression of Synaptophysin protein at day 50 of neuronal differentiation (n≥3). **B** Histogram of PSD-95 protein expression normalised to both GAPDH (n≥3). Data was analyzed using Students T-Tests and all data is presented as means ± SEM; *P<0.05, ***P<0.001, ****P<0.0001 vs Control. **C** Representative western blots of GAPDH, PSD-95 and MAP2 from a Control, Deletion and Duplication sample.
