# Supplementary material for: Using induced pluripotent stem cells to investigate human neuronal phenotypes in 1q21.1 deletion and duplication syndrome
Source: Mol Psychiatry. 2021 Jun 10;27(2):819–30. doi: 10.1038/s41380-021-01182-2 (PMC9054650; doi:10.1038/s41380-021-01182-2)
Supplement: Supplementary file 11 — Supplementary Figure 10 [file 41380_2021_1182_MOESM11_ESM.pdf]

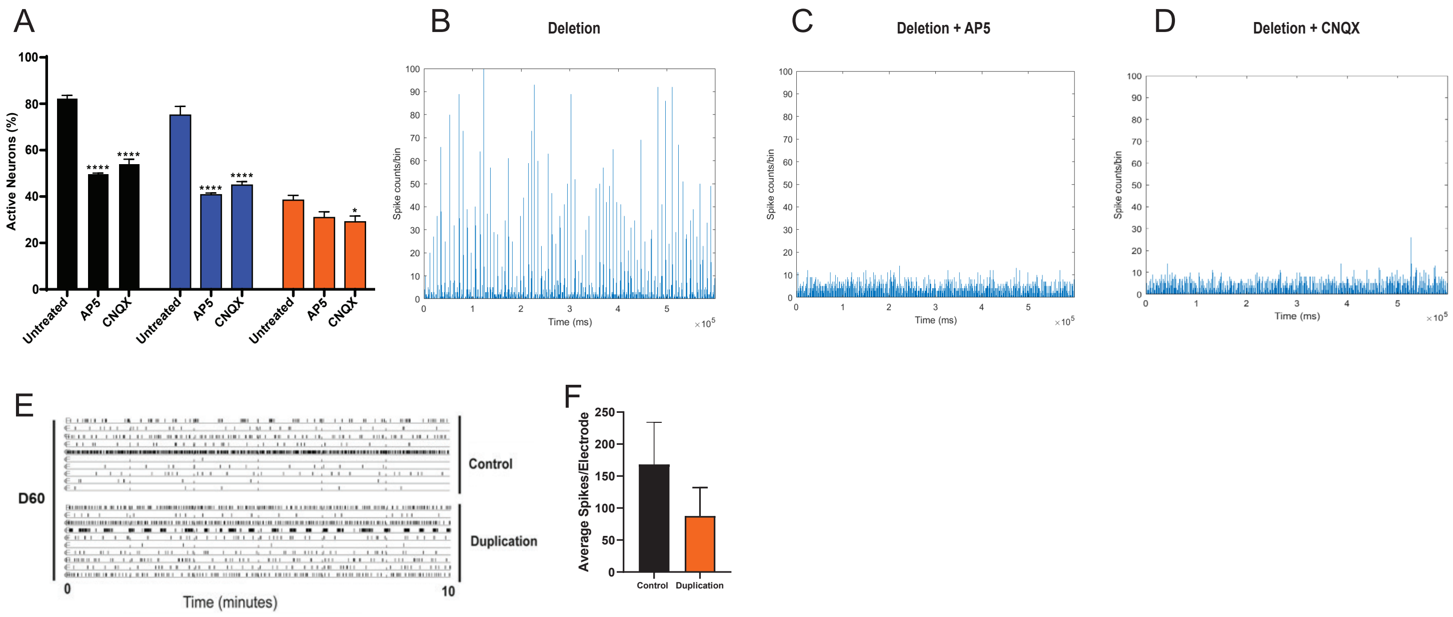

Supp. Fig. 10: Inhibition of AMPA or NMDA signalling results in decreased neuronal function. A Quantification of soma which show at least 1 characteristically neuronal calcium event (n≥3). Both genotype (F2,19=97.44; P<0.0001; n≥ 3/group) and drug (F2,19=100.5; P<0.05; n≥3/group) had significant effects on the percentage of active cells. There was also a significant interaction between the effect of genotype and drug (F4,19=10.16; P=0.0001; n≥3/group) on the percentage of active cells. Data sets were analysed by two-way ANOVA with post hoc comparisons using Dunnett's multiple comparisons test comparing to control samples. Stars represent Dunnett-corrected post hoc tests. All data presented as means ± SEM *P<0.05; ****P<0.0001 vs. untreated. **B** Example of an array-wide spike detection rate (ASDR) plot from 1q21.1 deletion cultures after 100 days of differentiation. **C** Example of an array-wide spike detection rate (ASDR) plot from 1q21.1 deletion cultures after 100 days of differentiation. The culture was incubated with AP5 immediatly before recording. **D** Example of an array-wide spike detection rate (ASDR) plot from 1q21.1 deletion cultures after 100 days of differentiation. The culture was incubated with CNQX immediatly before recording. **E** Representative raster plot of neuronal activity exhibited by control and duplication-derived neurons at early (D60) neurodevelopmental stage. **F** The average number of spikes recorded per electrode at D60 of differentiation in control and duplication-derived neurons, there was no significant difference between the spike counts in both models.
