# Supplementary material for: Using induced pluripotent stem cells to investigate human neuronal phenotypes in 1q21.1 deletion and duplication syndrome
Source: Mol Psychiatry. 2021 Jun 10;27(2):819–30. doi: 10.1038/s41380-021-01182-2 (PMC9054650; doi:10.1038/s41380-021-01182-2)
Supplement: Supplementary file 12 — Supplementary Figure 11 [file 41380_2021_1182_MOESM12_ESM.pdf]

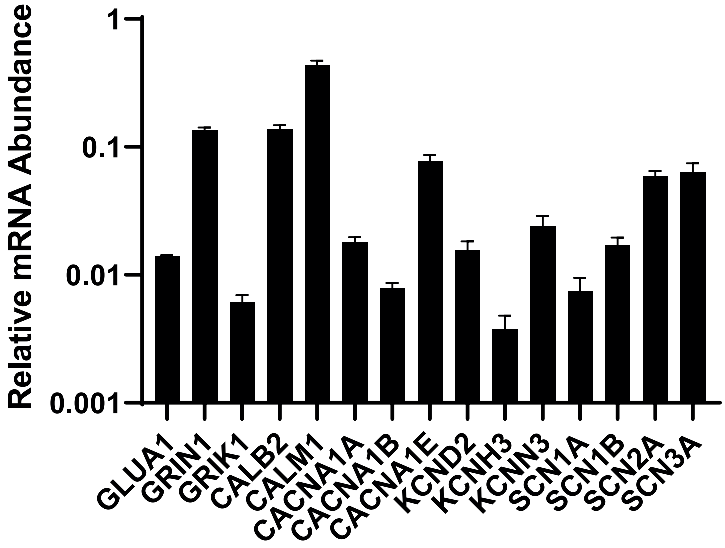

Supp. Fig. 11:Gene expression of ion channels in control day 50 neurons. Expression of common neuronal ion channels in day 50 control (average of both control, n≥3) neuronal culutres normalised to the expression of GAPDH.
