# Supplementary material for: Using induced pluripotent stem cells to investigate human neuronal phenotypes in 1q21.1 deletion and duplication syndrome
Source: Mol Psychiatry. 2021 Jun 10;27(2):819–30. doi: 10.1038/s41380-021-01182-2 (PMC9054650; doi:10.1038/s41380-021-01182-2)
Supplement: Supplementary file 13 — Supplementary Table 1 [file 41380_2021_1182_MOESM13_ESM.pdf]

Supp. Table 1: Patient information for all 1q21.1 patients used in the study. GAD: generalised anxiety disorder; MDD: major depressive; OCD: obsessive compulsive disorder; PD: personality disorder.

	Sex	Full scale IQ	Clinical Symptoms	Medication	1q21.1 Mutation Coordinates	Other CNVs
Deletion 1	male	77	agoraphobia, avoidant PD, obsessive-compulsive PD, depressive PD, other substance-related disorder, conduct disorder	N/A	chr1:146496661-147375981	chr12:2027625-2550818_dup
Deletion 2	female	78	Adjustment Disorder, cardiac: deformed valve, narrowing of aorta, thyroid problems, hearing difficulties, sleep apnoea	Citalopram	chr1:146330584-147825662	chr1:63918079-64038174_dup, chr5:19503096-19985234_del
Deletion 3	male	109	social phobia, OCD, MDD, avoidant PD; interstitial cystitis (daily catheterisation), short stature, recurrent infections; tremor	Propranolol	chr1:146496661-147391614	
Duplication 1	female	89	social phobia, GAD, MDD, avoidant PD, conduct disorder, hearing difficulties	N/A	chr1:146330584-146982763	
Duplication 2	female	109	social phobia, panic disorder, agoraphobia, OCD, MDD, psychotic symptoms, schizotypal PD, Borderline PD; osteoarthritis, visual difficulties;	Propranolol	chr1:146330584-147825662	
