# Supplementary material for: Using induced pluripotent stem cells to investigate human neuronal phenotypes in 1q21.1 deletion and duplication syndrome
Source: Mol Psychiatry. 2021 Jun 10;27(2):819–30. doi: 10.1038/s41380-021-01182-2 (PMC9054650; doi:10.1038/s41380-021-01182-2)
Supplement: Supplementary file 14 — Supplementary Table 2 [file 41380_2021_1182_MOESM14_ESM.pdf]

Supplementary Table 2: List of primers used in the study

Target	Forward	Reverse
BCL9	GGCCATACCCCTAAAGCACTC	CGGAAATACTTCGCTCCCTTTT
CACNA1A	CGCTTCGGAGACGAGATGC	TGCGCCATTGACTGCTTGT
CACNA1B	GACAACGTCGTCCGCAAATAC	CCCGATGAAATAGGGCTCCG
CACNA1E	AATGATGCCTTAGGAGCCACC	AGCTCACGCTCAATCTGCTG
CALB2	AGCGCCGAGTTTATGGAGG	TGGTTTGGGTGTATTCCTGGA
CALM1	TTGACTTCCCCGAATTTTTGACT	GGAATGCCTCACGGATTTCTT
CHD1L	GCTATGAGCGTGTGGATGGTT	TGCTGTTAAGTTCATGCCAACTC
CUX1	GCTCTCATCGGCCAATCACT	TCTATGGCCTGCTCCACGT
DCX	CCTTGGCTAGCAGCAACAGT	CCACTGCGGATGATGGTAA
ETV1	CTGGATGACCCGGCAAATTCT	CCTCTTCAGGCTCAATCAGTTT
FOXP1	AGACAAAAAGTAACGGTTCAGCC	CGCACTCTAGTAAGTGGTTGC
GFAP	AGGTCCATGTGGAGCTTGAC	GCCATTGCCTCATACTGCGT
GAPDH	CTGGTAAAGTGGATATTGTTGCCAT	TGGAATCATATTGGAACATGTAAACC
GLUA1	CGAGCTTTCCCGTTGATACAT	TCTGCCACTTGTAATGGTCAATG
GPR89B	GGAGTGACTCTCATGGCTCTT	TGTTATGCACTTCCCCCTTCT
GRIK1	TCCTCTGCTATATCCTCCCTCA	CATCAGGGTTCGGTTTCTGTTA
GRIN1	CTACCGCATACCCGTGCTG	GCATCATCTCAAACCACACGC
KCND2	GGGTTTTTCATTGCCGTCTCT	CACAGCATACCGCTCTCCA
KCNH3	TGGACGAGCACAAGGAGTTC	CGGTTCTTGGTTTCGCTGATG
KCNN3	GCCTTCTCCTACACACCCTC	CTCGGGCGATCAGGTACAG
MAP2	CTGCTTTACAGGGTAGCACAA	TTGAGTATGGCAAACGGTCTG
NESTIN	TCCAGAAACTCAAGCACCA	AAATTCTCCAGGTTCCATGC
NOTCH2NL	TGAGCCTTTGAAGCAGGAGG	AGATCCACATGGGGAGGGG
Pax6	CAACTCCATCAGTTCCAACG	TGGATAATGGGTTCTCTCAAACTCT
PRKAB2	ATGCGTTTCGATCTGAGGAAAG	GGTTCAGCATAACATGGTTGGG
PSD95	AGCCCCAGGATATGAGTTGC	GATGTGTGGGTTGTCAGTGC
REELIN	TCCGGGACAAGAATACCATGT	CCAAATCCGAAAGCACTGGAA
S100B	TGGCCCTCATCGACGTTTTC	ATGTTCAAAGAACTCGTGGCA
SATB2	CCGCACACAGGGATTATTGTC	TCCACTTCAGGCAGGTTGAG
SCN1A	ATGTGGAAATAGCTCTGATGCAG	AGCCCAACTGAAGGTATCAAAG
SYNAPTOPHYSIN	TGGTGTTCGGCTTCCTGAA	GCGGCCCAGCCTGTCT
TUJ1	CATGGACAGTGTCCGCTCAG	CAGGCAGTCGCAGTTTTCAC
