# Supplementary material for: Using induced pluripotent stem cells to investigate human neuronal phenotypes in 1q21.1 deletion and duplication syndrome
Source: Mol Psychiatry. 2021 Jun 10;27(2):819–30. doi: 10.1038/s41380-021-01182-2 (PMC9054650; doi:10.1038/s41380-021-01182-2)
Supplement: Supplementary file 15 — Supplementary Table 3 [file 41380_2021_1182_MOESM15_ESM.pdf]

Supplementary Table 3: List of antibodies used in the study.

Target	Supplier (code)	Host	Dilution
Oct4	CellSignalling (C30A3)	Rabbit	1:400
Sox2	CellSignalling (D6D9)	Rabbit	1:400
Nanog	CellSignalling (D73G4)	Rabbit	1:200
MAP2	R and D (MAB8304)	Mouse	1:1000
TBR1	Abcam (Ab31940)	Rabbit	1:200 - 1:1000
TUJ1	Sigma (T8578)	Mouse	1:500
CTIP2	Abcam (Ab18465)	Rat	1:200 - 1:1000
MAP2	Abcam (Ab32454)	Rabbit	1:1000
Synaptophysin	Abcam (Ab32127)	Rabbit	1:500
GAPDH	Abcam (ab9485)	Rabbit	1:5000
PSD95	Abcam (ab76115)	Rabbit	1:1000
Ki67	Abcam (ab15580)	Rabbit	1:1000
Nestin	Abcam (ab105389)	Rabbit	1:300
